# Fatty Acid Composition of Pseudocereals and Seeds Used as Functional Food Ingredients

**DOI:** 10.3390/life13010217

**Published:** 2023-01-12

**Authors:** Małgorzata Czerwonka, Agnieszka Białek

**Affiliations:** 1 Department of Bromatology, Faculty of Pharmacy, Medical University of Warsaw, Banacha 1, 02-097 Warsaw, Poland; 2 School of Health & Medical Sciences, University of Economics and Human Sciences in Warsaw, Okopowa 59, 01-043 Warsaw, Poland; 3 The Kielanowski Institut of Animal Physiology and Nutrition, Polish Academy of Sciences, Instytucka 3, 05-110 Jabłonna, Poland

**Keywords:** fatty acids profile, seeds, pseudocereals, functional ingredients

## Abstract

In recent times, the popularity of seeds, other than cereals, in the diet has systematically grown. The fat contained in these products significantly affects their energy value as well as their biological and physicochemical properties, including their susceptibility to oxidation. The objective of this study is to evaluate the fat concentration and fatty acid (FA) composition of popular non-spice seeds used in food as a substitute for cereals or a functional additive. The research material consisted of thirteen groups of seeds derived from the following plants: amaranth, blue poppy, buckwheat, chia, flax, hemp, canihua, milk thistle, pumpkin, plantago, quinoa, sesame, and sunflower. The fat contents and fatty acid profiles differed significantly between the tested products and were dependent on the plant species. In all products, polyunsaturated fatty acids (PUFAs, 40–80% of total FAs) dominated. Linoleic acid was the main FAs in most tested seeds. The exceptions were chia and flax seeds, which were characterized by very high contents of α-linolenic acid, respectively, 62.0 and 51.4% of the total FAs. The share of monounsaturated FAs (mainly oleic acid) in the total FAs content was between 6 and 40%. All tested seeds (especially flax, chia, and hemp) have favorable values for their indexes of atherogenicity and thrombogenicity as well as the hypocholesterolemic/hypercholesterolemic ratio.

## 1. Introduction

Seeds have played an important role in human nutrition since the Palaeolithic times. Nowadays, they, especially those from cereal plants, are the basis of most people’s diets around the world. The seed is the organ of a plant from which a new organism is formed, and, within it, there is a store of nutrients for the seedling that will grow from the embryo. Therefore, depending on the plant species, seeds are the source of starch, fat and fat-soluble vitamins, proteins, dietary fiber, minerals, or flavor ingredients [1,2,3].

In recent times, the popularity of seeds other than cereals in the diet has systematically grown. They are used as functional ingredients for bakery and confectionery products, milk-based products, salads, and many more food items [4,5]. They are used mainly due to their sensory qualities and high content of fiber or fat, in which polyunsaturated fatty acids (PUFAs) dominate. The fatty acid (FA) composition in seed oil can significantly affect the physicochemical and biological properties of these products [6,7]. Particular attention should be paid to seeds potentially containing significant amounts of α-linolenic acid (ALA), such as chia, flax, and hemp [8,9]. 

With the increasing popularity of gluten-free diets, pseudocereals, i.e., plants that do not belong to grasses but whose seeds are rich in starch, have gained popularity. The most common are buckwheat, amaranth, quinoa, and canihua [10]. Chia is also often considered a pseudocereal, but the starch content in the seeds of this species is rather small compared to the abovementioned ones [11]. They do not contain gluten, so, for people with celiac disease or those on a gluten-free diet for other reasons (right or wrong), these seeds, in the form of groats or flour, are an alternative to cereals [12]. When cereal products are completely replaced with pseudocereal products, the fat contained in them (even with a small percentage share) can be a significant part of total dietary lipid intake [13].

Seeds are not a homogeneous group of food products. The nutrient contents, including fat and fatty acids, vary between species. Despite the huge interest in seeds as functional ingredients or cereals mimetics, there are very few papers that compare the content and profile of FAs in these products. The present study aims to evaluate the FAs profile of popular non-spice seeds used in food as a substitute for cereals or functional additives. 

## 2. Materials and Methods

### 2.1. Research Material

The research material comprised thirteen groups of plant seeds; pseudocereals and seeds were used as functional food ingredients (fiber and/or PUFAs source). Spices, legumes seeds, and nuts were not examined in this work. All groups of tested products are listed in Table 1. Each group contains eight products from different brands. All food samples were purchased at a local market in Warsaw, Poland. 

### 2.2. Analytical Methods

Fat content in seed samples was determined gravimetrically after extraction with a mixture of chloroform/methanol (*v/v* 2:1) and solvent evaporation under a stream of nitrogen, according to the procedure described by Folch and co-workers [14].

The FAs profile of the examined seeds was determined as FAME by gas chromatography. The extracted fat was saponified by 0.5 M NaOH in methanol, followed by FAs methylation using a 14% boron trifluoride–methanol reagent. The FAME were isolated with hexane after adding saturated sodium chloride solution. The procedure was based on the AOAC method [15], previously described by Białek and co-workers [16]. Analyses were performed on a gas chromatograph with a flame ionization detector (Shimadzu GC-17A, Kyoto, Japan). Chromatographic separations were conducted on a capillary column SGE BPX70 (60 m/0,25 mm ID/film thickness 0, 20 μm; Ringwood, Australia). Helium was used as the carrier gas (flow: linear velocity at 0.9 mL min^−1^), the injection was 1 μL, and the split was set to 10. The injector was heated to 250 °C, and the detector to 270 °C. The temperature program was as follows: initial oven temperature at 140 °C for 1 min, increase by 20 °C per min to 200 °C, hold for 20 min, increase by 5 °C per min to 220 °C, and hold for 25 min. FAME standards (Supelco 37 FAME Mix No. 47885-U) were used to identify the FAs present in the samples. Results are expressed as a percentage of total FAs content. All chemical analyses were carried out in triplicate.

Based on the percentage of fatty acids, the index of atherogenicity (IA), the index of thrombogenicity (IT), and the hypocholesterolemic/hypercholesterolemic ratio (H/H) were calculated as follows [17]:

IA = [C12:0 + (4 × C14:0) + C16:0]/(MUFA + n3 PUFA + n6 PUFA)

IT = [C14:0 + C16:0 + C18:0]/[(0.5 × MUFA) + (0.5 × n6 PUFA) + (3 × n3 PUFA) +

(n3 PUFA/n6 PUFA)]

H/H = (C18:1 + C18:2 LA + C18:3 ALA)/(C12:0 + C14:0 + C16:0)

### 2.3. Statistical Analyses

Data are expressed as mean values ($\bar{x}$) and standard deviation (SD). Because variables in groups were normally distributed, as assessed by the Shapiro–Wilk test, the data were analyzed by one-way ANOVA (α = 0.01), followed by Tukey’s test (α = 0.05); the same superscript letters (next to the results) indicate homogeneous groups. In addition, a cluster analysis of FAs profiles was performed in which only FAs, present in all examined groups, were used. Differences in the FAs profiles among clusters were verified with the Ward agglomeration procedure as a grouping method, and the Euclidean distance, as a function of the distance, was used. The cut-off point was established at 33% of the maximum distance, according to Sneath’s criterion. All results were evaluated using Statistica 13.3 software (StatSoft, Kraków, Poland).

## 3. Results

The fat contents and FAs profiles varied significantly among the tested seeds. The obtained results are presented in Table 2. The lowest lipid concentration was determined in plantago seeds. The fat content in pseudocereals (BW, Q, A, and K) varied between 2.7 and 8.0 g·100 g^−1^; there were no statistically significant differences among the species of these plants. In the other examined seeds used as functional products or food ingredients (except PP), the lipid concentration was significantly higher than in pseudocereals, and its levels were from 20 to over 50 g·100 g^−1^. The highest content of lipids was determined in sesame and sunflower seeds. 

In all tested products, PUFAs dominated, and the share of saturated FAs (SFAs) was the lowest (except C). Nevertheless, the proportions between the main FAs groups were strongly dependent on the seed species and varied greatly among the different products (Table 3). 

The SFAs concentrations in the examined seeds ranged from 9% (H, SN) to 25% of the total FAs (A). In all samples, palmitic (C16:0), stearic (C18:0), and arachidic (C20:0) acids were present. Palmitic acid was the dominant SFAs in all seeds. Among FAs with an even number of carbons in the chain, lauric acid (C12:0) was detected only in PP seeds. The presence of myristic acid (C14:0) was not confirmed in C, L, BP, H, S, and SN, whereas, in other examined seeds, a significantly higher content was detected in PP. The share of behenic acid (C22:0) in the total FAs pool was the highest in SM and Q; its small share was typical for L, P, and S, whereas, in C, BP, and H, the presence of C22:0 was not confirmed. There were only two FAs with an odd number of carbons in their chain present in the examined samples: C21:0 and C23:0; of these, C21:0 was detected only in BW and K. 

The share of monounsaturated FAs (MUFAs) in the total FAs content was from 6% (C) to 40% (S). Oleic acid (C18:1) was the main MUFAs. Moreover, it was the only MUFAs that was determined in all examined seeds; in S and L, it was the only detected one. Other FAs with an even number of carbons in the chain analyzed in this study were: C14:1, which was specific for PP seeds; C16:1; C20:1; and C22:1 n9. Palmitoleic acid (C16:1) was not present in SM and L, and, among the other examined seeds, its highest share was found in PP. C20:1 was specific only for Q and H, whereas C22:1 n9 was typical solely for H. C17:1 was the only MUFA with an odd number of carbons in the chain and was present in four examined seeds (PP, H, A, and K), but its highest share was confirmed for A seeds. 

The PUFAs content ranged from 40% (BW, S) to over 80% (C) of the total FAs. Linoleic (LA, C18:2 n6), α-linolenic (ALA, C18:3 n3), and γ-linolenic (C18:3 n6) acids were detected in all samples. The highest concentration of LA was detected in BP; its share was about 70% of the total FAs. However, LA was the dominant FAs in most of the examined seeds, such as SN, H, and SM, in which it constituted over 50% of the total FAs. ALA predominated in the total FAs in two species of seeds: in C, its content was 62%, and, in L, its content was about 51% of the total FAs. Some other detected PUFAs were characteristic only for selected seeds: C20:2 for PP and H, C20:3 n6 for K, C20:3 n3 for PP, and C20:4 n6 for H. Surprisingly, the presence of C20:5 n3 was established in five examined types of samples, but its share was the highest in H seeds (1.34 ± 0.09%). A significantly higher content of C20:2 was typical for A seeds, in which it exceeded 2%. 

The indexes of atherogenicity and thrombogenicity and the hypocholesterolemic/hypercholesterolemic ratio result from the fatty acid composition. The most advantageous parameters, a low IA and IT and a high H/H, are those of flax seed, for which the IA, IT, and H/H were, respectively, 0.09, 0.06, and 14.3. Chia and hemp oil were not much different from linseed. Amaranth seeds are characterized by the highest IA and IT and the lowest H/H ratio. The results are presented in Table 3.

**Figure 1:**
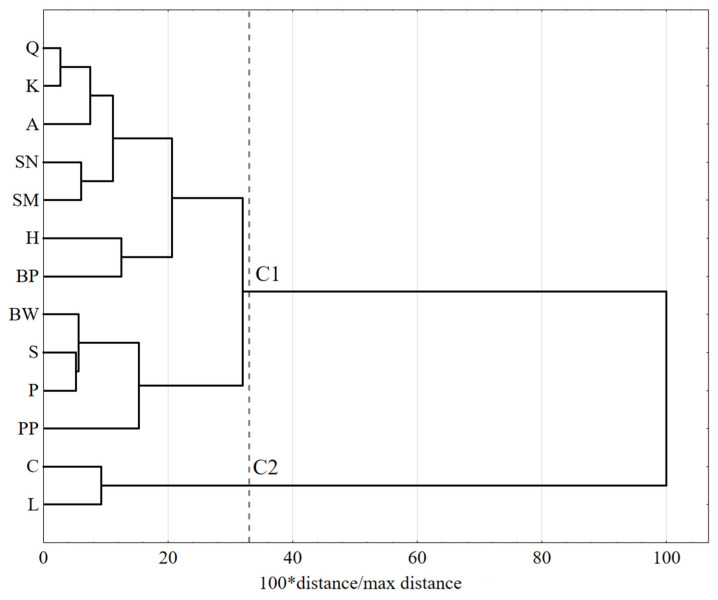
Dendrogram of similarity in fatty acid profiles of selected seeds. C1–C2—clusters. A—amaranth; BP—blue poppy; BW—buckwheat; C—chia; H—hemp; K—canihua; L—flax; P—pumpkin; PP—plantago; Q—quinoa; S—sesame; SM—milk thistle; SN—sunflower.

A multivariate statistical procedure (cluster analysis) was applied to determine similarities in the FAs profiles among examined seeds. A cluster analysis of the FAs profiles of the tested products revealed two clusters: C1 and C2 (Figure 1); the content of oleic acid (*p* = 0.036), LA (*p* = 0.001), and ALA (*p* < 0.0001) caused the distinction between clusters. Cluster C2 includes only two seeds: L and C. Both of these seeds were characterized by a significantly higher content of ALA and lower levels of LA and OL compared to other products (*p* = 0.0002). All other examined products were in cluster C1. The content of SFAs (palmitic, stearic, and arachidic acids) did not affect allocations to the clusters.

## 4. Discussion

There is convincing evidence that the consumption of seeds has beneficial effects on human health [2]. These properties may partly result from the content of lipid components. Although fat from the seeds of studied plants is composed not only of FAs but also many other bioactive ingredients, knowledge of the FAs compositions indicates their biological and physicochemical properties, including their susceptibility to oxidation and the possibility of using seeds, or the oils obtained from them, as food additives, components of dietary supplements, or cosmetics [18].

Unsaturated FAs predominated in all tested seeds, which is characteristic of plant oils. However, the fat contents and FAs compositions in these seeds strongly depended on the plant species. In the multivariate statistical procedure, based on the content of FAs present in all examined products, only two clusters were distinguished. In the first cluster, there were seeds with a higher level of ALA and lower LA and oleic acid contents. The next cluster contained all of the other products. However, in most examined seeds, many other FAs have been identified, whose contents were unique to the given plant.

Due to the high content of unsaturated fatty acids, the tested seeds were characterized by very favorable values for the indexes of atherogenicity (IA) and thrombogenicity (IT) and the hypocholesterolemic/hypercholesterolemic ratio (H/H), compared to other foods, especially those of animal origin [17,19]. The abovementioned indexes are used to evaluate the nutritional value of the fat. Low and very low IA and IT and high H/H ratios testify to the potentially beneficial effect of fat from the tested seeds on the functioning of the cardiovascular system [20].

Most of the tested seeds (sunflower, poppy, sesame, and pumpkin) are widely used in the bakery and confectionery industry [4]. The oils from these are equally popular. Refined sunflower oil is often used for frying. Cold-pressed pumpkin or sesame oils, due to their characteristic organoleptic properties, are popular as food additives [21]. Moreover, pumpkin seed oil is rich in phenolic compounds and phytosterols [22], whereas sesame oil contains abundant lignans, including sesamin, sesamolin, and lignan glycosides [23]. Milk thistle seeds are used primarily as a source of dietary fiber, but seed oil is also a very valuable resource due to its high content of silymarin compounds [24]. Plant seeds are a source of valuable fat with comprehensive properties that are the result of the FAs composition and content of the bioactive components.

The results of the fat contents and FAs compositions of all of the abovementioned seeds coincided with the data presented by other authors. However, they often emphasize that these parameters may vary significantly depending on the region of cultivation, weather conditions, and variety [25,26,27,28].

### 4.1. Seeds as a Source of Essential n-3 Polyunsaturated Fatty Acids

The amount and quality of fat compounds supplied with food to our bodies are critical to maintaining good health. In developed countries, the problem is a too-high supply of fat and SFAs in the diet, as well as a too-high ratio of n6 PUFAs to n3 PUFAs [29]. A disordered relationship between these two families of FAs may result in impaired immune balance, increased propensity to inflammation, and, in the long term, an increased risk of many non-communicable diseases [30]. Because some seeds can be an excellent source of ALA, by including them in the diet, we can greatly improve the ratio between n6 PUFAs and n3 PUFAs [31]. 

Two species of seeds were distinguished by a high content of n3 PUFAs. The direct content of ALA in chia and flax seeds is comparable (>18%), but, taking into account the fat extracted from these seeds, the higher content of this essential FAs is in chia oil. In the present work, the ALA concentration in chia seeds was 62% of the total FAs, which is consistent with the work of other authors, in which the level of this acid ranged from 52 to 69% [32,33] and varied depending on the area of cultivation (growing conditions) [34]. The content of ALA in linseed oil determined in this study was also consistent with data from the literature, according to which the concentration of this component ranges from 51 to 63% of the total FAs. Other authors point out that, as in the case of chia, growing conditions and cultivar have a significant impact on the oil content and FAs profile of linseed oil [8,35].

ALA is a precursor of the long-chain PUFAs metabolically formed in humans. There is conclusive evidence that regular consumption of flaxseed and chia seeds, as well as products obtained from them, brings beneficial effects to the body. The meta-analysis, carried out by Ursoniu and co-workers [36], showed significant reductions in both systolic and diastolic blood pressure following supplementation with various flax seed products. Other studies suggested that flaxseed oil in high doses suppresses inflammatory mediators, decreases platelet aggregation, and increases bleeding time [37,38]. In addition, cholesterol-lowering actions have been demonstrated for these seeds [39]. Animal and human studies have also shown a positive effect of chia seed intake in a diet, which includes a reduction in cholesterol and systolic blood pressure, inhibition of blood clotting, and anti-inflammatory properties [40]. Intake recommendations for ALA are about 1% energy, corresponding to 2–3 g of ALA per day [41]. To cover 100% of the daily requirement, one should consume from 11 to 16 g of chia or flax seeds (depending on the total energy content). It should also be emphasized that seeds are not the only ALA source in the diet. Flax and chia seeds are also successfully used in animal nutrition to obtain products (milk, meat, and eggs) with higher contents of n3 PUFAs [42,43,44].

An interesting composition of FAs is also evident in hemp seeds. According to the literature, the LA concentration in hemp oil ranges from 50 to 70% of the total FAs, whereas the ALA level is 12–25% [45]. The proportion of these FAs is different than in fat from flax or chia seeds. The 3:1 ratio of LA to ALA (3.2:1 in the present study) is alleged to be optimal for nutrition [46]. 

Flax, chia, and hemp seed oils are widely available for sale. However, fats with a high content of n3 PUFAs are more susceptible to oxidation [47]; therefore, special attention should be paid to protecting these products (such as proper packaging and information on product storage) from the effects of temperature and sunlight to slow down the autoxidation processes.

### 4.2. Pseudocereals as a Source of Fat and Fatty Acids

The cultivation and consumption of pseudocereals have very long histories. Plants of the family Amaranthaceae were the most important crops in pre-Columbian America. After the conquest of these lands by the Spanish, the production and importance of these plants decreased [13]. For many years, the cultivation of buckwheat, which originally came from Asia, also declined [48]. However, in recent times, pseudocereals have gained increasing importance. This is due to the popularity of gluten-free diets. It is estimated that 1% of the world’s population suffers from celiac disease, gluten intolerance, and gluten ataxia. Seeds from pseudocereals and their products are a response to the needs of a large group of consumers who, for various reasons, do not consume gluten-containing food [49].

The fat content in pseudocereal seeds is small, with the lowest determined for buckwheat. There is little information about the content and composition of FAs in buckwheat seeds. The few available data confirm the results obtained in the present work [50,51]. This product is of interest mainly due to the content of non-fat bioactive components, primarily polyphenols: rutin, orientin, vitexin, quercetin, isovitexin, and isoorientin [52]. However, lipid components may affect the technological properties of seeds and the flour obtained from them, as well as the oxidative stability of these products. 

The fat contents and FAs compositions in seeds of plants from the Amaranthaceae family (quinoa, canihua, and amaranth) were quite similar. According to other authors, the lipid levels of seeds range from 4 to 8.3 g·100 g^−1^, which agrees with the results obtained by this study [53,54]. The FAs composition in this group of pseudocereal seeds is very similar to the cereal grains. The FAs profile presented in this paper also corresponds with the works of other authors [55]. 

The range of quinoa, canihua, and amaranth products is huge; however, oil, on an industrial scale, is obtained mainly from amaranth as a byproduct of the production of high-protein amaranth flour or protein and starch isolations [56]. Amaranth oil is rich in squalene, and this component is widely used in pharmaceutical and cosmetic applications [57]. A significant amount of squalene can also be found in other species of plants from this family [58].

Amaranth, quinoa, and buckwheat lipids are characterized by a high degree of unsaturation, which is desirable from a nutritional point of view. Moreover, they are quite resistant to oxidation, which is of great importance during the technological processes to which they are subjected [13].

## 5. Conclusions

Seeds are a source of fat and essential unsaturated fatty acids in the human diet. However, fat content and FAs composition depend on the species of plant from which the seeds were obtained. In most tested products, the predominant acid is LA, which belongs to the n6 family. Only in flax and chia seeds, ALA is the main FAs. These two products stand out because of the FAs composition. Consuming even small amounts of flax or chia seeds in the diet can supplement the deficiencies of n3 PUFAs. Due to the high content of unsaturated fatty acids, all examined products characterize a beneficial index of atherogenicity, index of thrombogenicity, and hypocholesterolemic/hypercholesterolemic ratio.

## Figures and Tables

**Table 1:** Research material; tested plant seeds.

Name	Family	Abbreviation
Pseudocereals:		
amaranth (*Amaranthus cruentus* L.)	*Amaranthaceae*	A
buckwheat (*Fagopyrum esculentum*)	*Polygonaceae*	BW
canihua/kaniwa (*Chenopodium pallidicaule*)	*Amaranthaceae*	K
quinoa (*Chenopodium quinoa*)	*Amaranthaceae*	Q
Food functional ingredients:		
chia (*Salvia hispanica*)	*Lamiaceae/Labiatae*	C
flax (*Linum usitatissimum* L.)	*Linaceae*	L
hemp (shelled seeds; *Cannabis sativa* L.)	*Cannabaceae*	H
milk thistle (*Silybum marianum)*	*Asteraceae*	SM
plantago (*Plantago psyllium)*	*Plantaginaceae*	PP
poppy (blue, *Papaver somniferum)*	*Papaveraceae*	BP
pumpkin (shelled seeds, *Cucurbita* L.)	*Cucurbitaceae*	P
sesame (shelled seeds, *Sesamum indicum* L.)	*Pedaliaceae*	S
sunflower (shelled seeds, *Helianthus annuus*)	*Asteraceae*	SN

**Table 2:** Fat contents and fatty acid compositions of selected seeds ($\bar{x}$ ± SD; *n* = 8 per group).

	PP	BW	Q	A	K	SM	C	L	P	BP	H	S	SN	*p* Value *
Fat [g 100 g ^−1^]														
	1.2 ± 1.2 ^a^	2.9 ± 0.2 ^a,b^	6.0 ± 1.1 ^b^	6.1 ± 0.6 ^b^	7.3 ± 0.7 ^b^	23.7 ± 3.2	29.4 ± 1.4	36.0 ± 2.0 ^c^	38.0 ± 5.9 ^c,d^	41.0 ± 3.9 ^d^	41.6 ± 3.1 ^d^	49.1 ± 3.1 ^e^	51.7 ± 3.1 ^e^	<0.0001
SFAs [% of total FAs]														
C12:0	0.17 ± 0.04	nd	nd	nd	nd	nd	nd	nd	nd	nd	nd	nd	nd	-
C14:0	0.35 ± 0.08	0.13 ± 0.00 ^a^	0.17 ± 0.05 ^a,b^	0.23 ± 0.03 ^b^	0.15 ± 0.01 ^a^	0.11 ± 0.01 ^a^	nd	nd	0.12 ± 0.02 ^a^	nd	nd	nd	nd	<0.0001
C16:0	15.1 ± 2.4 ^e^	15.3 ± 1.0 ^e^	10.4 ± 0.7 ^c^	19.9 ± 2.2	14.2 ± 0.3 ^d,e^	8.62 ± 0.36 ^b,c^	7.40 ± 0.21 ^a,b^	6.17 ± 0.39 ^a^	12.5 ± 1.1 ^d^	9.65 ± 0.61 ^c^	6.33 ± 0.60 ^a^	9.73 ± 0.31 ^c^	6.26 ± 0.40 ^a^	<0.0001
C18:0	2.71 ± 0.28 ^d^	1.66 ± 0.20 ^b^	0.42 ± 0.08 ^a^	3.03 ± 0.52 ^d^	0.86 ± 0.19 ^a^	4.96 ± 0.44 ^e^	2.95 ± 0.56 ^d^	3.99 ± 0.55	6.10 ± 0.38 ^f^	1.93 ± 0.19 ^b,c^	2.68 ± 0.28 ^c,d^	5.57 ± 1.03 ^e,f^	2.44 ± 0.29 ^c,d^	<0.0001
C20:0	0.55 ± 0.08 ^d,e,f^	1.33 ± 0.19	0.43 ± 0.08 ^c,d,e^	0.72 ± 0.06 ^f^	0.60 ± 0.02 ^e,f^	2.70 ± 0.38	0.25 ± 0.03 ^a,b,c^	0.16 ± 0.02 ^a,b^	0.38 ± 0.03 ^b,c,d^	0.11 ± 0.01 ^a^	0.70 ± 0.10 ^f^	0.58 ± 0.05 ^d,e,f^	0.21 ± 0.02 ^a,b^	<0.0001
C21:0	nd	0.17 ± 0.01 ^a^	nd	nd	0.12 ± 0.01 ^a^	nd	nd	nd	nd	nd	nd	nd	nd	<0.0001
C22:0	0.67 ± 0.14 ^c^	1.56 ± 0.27	0.61 ± 0.08 ^c^	0.32 ± 0.04 ^a,b^	0.45 ± 0.02 ^b,c^	1.82 ± 0.28	nd	0.12 ± 0.01 ^a^	0.12 ± 0.01 ^a^	nd	nd	0.11 ± 0.01 ^a^	0.66 ± 0.08 ^c^	<0.0001
C23:0	0.27 ± 0.09 ^a,b^	0.19 ± 0.03 ^a^	0.36 ± 0.20 ^b^	0.44 ± 0.06 ^a^	0.19 ± 0.05 ^a^	0.18 ± 0.00 ^a^	nd	nd	0.13 ± 0.01 ^a^	nd	nd	nd	0.12 ± 0.02 ^a^	<0.0001
MUFAs [% of total FAs]														
C14:1	0.24 ± 0.05	nd	nd	nd	nd	nd	nd	nd	nd	nd	nd	nd	nd	-
C16:1	0.47 ± 0.17	0.22 ± 0.01 ^b,c^	0.14 ± 0.02 ^a,b^	0.31 ± 0.03 ^c^	0.10 ± 0.00 ^a^	nd	0.17 ± 0.01 ^a,b^	nd	0.15 ± 0.06 ^a,b^	0.14 ± 0.03 ^a,b^	0.11 ± 0.01 ^a,b^	0.12 ± 0.01 ^a,b^	0.12 ± 0.02 ^a,b^	<0.0001
C17:1	0.19 ± 0.04 ^a^	nd	0.21 ± 0.03 ^a,b^	0.71 ± 0.05	0.26 ± 0.04 ^b^	nd	nd	nd	nd	nd	nd	nd	nd	<0.0001
C18:1	28.7 ± 3.3 ^f,g^	33.7 ± 0.9 ^g,h^	24.1 ± 3.9 ^d,e,f^	21.1 ± 0.5 ^c,d,e^	24.0 ± 0.7 ^d,e,f^	24.4 ± 3.3 ^e,f^	5.64 ± 0.76 ^a^	17.3 ± 1.1 ^b,c,d^	33.2 ± 8.0 ^g^	16.6 ± 2.9 ^b,c^	11.3 ± 3.0 ^a,b^	40.0 ± 1.7 ^h^	33.7 ± 9.3 ^g,h^	
C20:1	nd	nd	0.09 ± 0.00	nd	nd	nd	nd	nd	nd	nd	0.76 ± 0.61	nd	nd	0.347
C22:1 n9	nd	nd	nd	nd	nd	nd	nd	nd	nd	nd	0.26 ± 0.06	nd	nd	-
PUFAs [% of total FAs]														
C18:2 n6	28.4 ± 3.9	37.5 ± 1.2 ^b^	50.4 ± 3.7 ^d,e^	45.8 ± 1.0 ^c,d^	48.9 ± 0.4 ^c,d,e^	55.1 ± 4.0 ^e^	19.6 ± 0.5 ^a^	19.1 ± 6.7 ^a^	46.6 ± 7.2 ^c,d^	69.2 ± 3.5	56.2 ± 1.4 ^e^	41.6 ± 2.1 ^b,c^	54.4 ± 8.8 ^e^	<0.0001
C18:3 n6	nd	nd	0.10 ± 0.00 ^a^	0.14 ± 0.04 ^a^	nd	nd	0.21 ± 0.01 ^b^	0.18 ± 0.02 ^b^	nd	nd	0.50 ± 0.00	nd	nd	<0.0001
C18:3 n3	14.5 ± 1.4 ^c^	2.12 ± 0.13 ^a,b^	5.78 ± 1.82 ^b^	0.83 ± 0.02 ^a^	5.06 ± 0.15 ^b^	0.22 ± 0.05 ^a^	62.0 ± 1.3	51.4 ± 6.7	0.24 ± 0.07 ^a^	0.71 ± 0.12 ^a^	17.3 ± 2.4 ^c^	0.33 ± 0.03 ^a^	0.25 ± 0.13 ^a^	<0.0001
C20:2 n6	0.18 ± 0.12	nd	0.14 ± 0.02	nd	nd	nd	nd	nd	nd	nd	nd	nd	nd	0.416
C20:3 n6	nd	nd	nd	nd	0.20 ± 0.01	nd	nd	nd	nd	nd	nd	nd	nd	-
C20:4 n6	nd	nd	0.22 ± 0.05	nd	nd	nd	nd	nd	nd	nd	nd	nd	nd	-
C20:3 n3	0.27 ± 0.08	nd	nd	nd	nd	nd	nd	nd	nd	nd	nd	nd	nd	-
C20:5 n3	0.13 ± 0.00 ^a^	0.13 ± 0.00 ^a^	1.34 ± 0.09	nd	0.74 ± 0.03	nd	nd	nd	nd	nd	0.26 ± 0.06	nd	nd	<0.0001
C22:2	nd	nd	0.65 ± 0.15 ^a^	2.85 ± 1.26	0.78 ± 0.24 ^a^	nd	nd	nd	0.12 ± 0.01 ^a^	nd	nd	nd	0.18 ± 0.00 ^a^	<0.0001

A—amaranth; BP—blue poppy; BW—buckwheat; C—chia; H—hemp; K—kaniwa; L—flax; P—pumpkin; PP—plantago; Q—quinoa; S—sesame; SM—milk thistle; SN—sunflower; FAs—fatty acids; SFAs—saturated fatty acids; MUFAs—monounsaturated fatty acids; PUFAs—polyunsaturated fatty acids; nd—not detected. * Statistically significant difference (α = 0.01). ^a–h^ Homogeneous groups in rows (Tukey’s test; α = 0.05); no superscript means that the result is significantly different from the other groups.

**Table 3:** Main groups of fatty acids [% of total FAs] and nutritional indexes for fatty acids ($\bar{x}$ ± SD; *n* = 8 per group).

	PP	BW	Q	A	K	SM	C	L	P	BP	H	S	SN	*p* Value *
SFAs	21.3 ± 2.6 ^a,b^	21.6 ± 1.7 ^a^	13.0 ± 1.0 ^e^	25.5 ± 2.4	17.2 ± 0.3 ^c,d^	18.6 ± 1.2 ^c^	10.8 ± 0.7 ^e,f^	10.6 ± 0.9 ^f^	19.3 ± 1.1 ^b,c^	11.9 ± 0.8 ^f^	10.2 ± 0.8 ^f^	16.3 ± 1.0 ^d^	9.82 ± 0.52 ^f^	<0.0001
MUFAs	32.0 ± 3.5 ^b,c^	36.1 ± 0.8 ^a,b^	25.7 ± 3.9 ^c,d^	23.0 ± 0.7 ^d,e^	25.2 ± 0.6 ^c,d^	24.9 ± 3.3 ^d^	5.92 ± 0.78 ^g^	17.6 ± 1.2 ^e,f^	33.5 ± 8.1 ^b^	17.1 ± 2.9 ^e,f^	12.8 ± 2.6 ^f,g^	40.9 ± 1.5 ^a^	34.5 ± 9.4 ^a,b^	<0.0001
PUFAs	46.7 ± 5.4 ^e,f^	42.3 ± 1.6 ^f^	61.3 ± 3.3 ^c^	51.5 ± 1.9 ^d,e^	57.6 ± 0.7 ^c,d^	56.6 ± 4.2 ^c,d^	83.3 ± 1.5 ^a^	71.8 ± 1.9 ^b^	47.2 ± 7.3 ^e,f^	71.1 ± 3.5 ^b^	77.1 ± 3.1 ^a,b^	42.8 ± 2.3 ^f^	55.7 ± 9.1 ^c,d^	<0.0001
n3 PUFAs	15.95 ± 1.6 ^a^	2.33 ± 0.19 ^c,d^	7.51 ± 1.90 ^b^	0.87 ± 0.03 ^d^	6.00 ± 0.17 ^b,c^	0.22 ± 0.06 ^d^	63.2 ± 1.3	52.3 ± 6.7	0.24 ± 0.07 ^d^	0.72 ± 0.12 ^d^	18.2 ± 2.9 ^a^	0.33 ± 0.03 ^d^	0.09 ± 0.15 ^d^	<0.0001
n6 PUFAs	30.8 ± 4.5	40.0 ± 1.4 ^d^	53.8 ± 4.1 ^a–c^	50.6 ± 1.9 ^b,c^	51.6 ± 0.6 ^a–c^	56.3 ± 4.2 ^a,b^	20.1 ± 0.6 ^e^	19.6 ± 6.8 ^e^	47.0 ± 7.3 ^c,d^	70.4 ± 3.6	58.9 ± 0.3 ^a^	42.4 ± 2.3 ^d^	55.6 ± 9.1 ^a,b^	<0.0001
n3/n6 PUFAs	0.52 ± 0.08 ^b^	0.06 ± 0.00 ^b,c^	0.14 ± 0.04 ^b,c^	0.02 ± 0.00 ^c^	0.12 ± 0.00 ^b,c^	0.00 ± 0.00 ^c^	3.14 ± 0.11 ^a^	2.97 ± 1.01 ^a^	0.01 ± 0.00 ^c^	0.01 ± 0.00 ^c^	0.31 ± 0.05 ^b,c^	0.01 ± 0.00 ^c^	0,00 ± 0.00 ^c^	<0.0001
IA	0.37 ± 0.05 ^c^	0.44 ± 0.03 ^b,c^	0.36 ± 0.05 ^c^	0.90 ± 0.12	0.49 ± 0.01 ^a,b^	0.36 ± 0.05 ^c^	0.11 ± 0.00 ^e,f^	0.09 ± 0.01 ^f^	0.42 ± 0.16 ^c^	0.56 ± 0.07 ^a^	0.21 ± 0.02 ^d,e^	0.24 ± 0.01 ^d^	0.20 ± 0.05 ^d–f^	<0.0001
IT	0.25 ± 0.04 ^d,e^	0.40 ± 0.04 ^a,b^	0.19 ± 0.02 ^f^	0.61 ± 0.08	0.28 ± 0.01 ^d^	0.34 ± 0.02 ^c^	0.05 ± 0.00 ^g^	0.06 ± 0.01 ^g^	0.46 ± 0.03 ^a^	0.26 ± 0.02 ^d^	0.11 ± 0.02 ^g^	0.37 ± 0.03 ^b,c^	0.20 ± 0.01 ^e,f^	<0.0001
H/H	4.75 ± 0.93 ^e,f^	4.77 ± 0.39 ^e,f^	7.87 ± 0.67 ^c^	3.57 ± 0.49 ^f^	5.56 ± 0.13 ^d,e^	9.23 ± 0.49 ^b^	11.8 ± 0.41	14.3 ± 1.1 ^a^	6.41 ± 0.61 ^d^	9.00 ± 0.61 ^b,c^	13.5 ± 1.3 ^a^	8.44 ± 0.30 ^b,c^	14.2 ± 1.1 ^a^	<0.0001

A—amaranth; BP—blue poppy; BW—buckwheat; C—chia; H—hemp; K—kaniwa; L—flax; P—pumpkin; PP—plantago; Q—quinoa; S—sesame; SM—milk thistle; SN—sunflower; SFAs—saturated fatty acids; MUFAs—monounsaturated fatty acids; PUFAs—polyunsaturated fatty acids; n3/n6 PUFAs—n3 and n6 PUFAs ratio; IA—index of atherogenicity; IT—index of thrombogenicity; H/H—hypocholesterolemic/hypercholesterolemic ratio. * Statistically significant difference (α = 0.01). ^a–g^ Homogeneous groups in rows (Tukey’s test; α = 0.05); no superscript means that the result is significantly different from the other groups.

## Data Availability

The data presented in this study are available on request from the corresponding author.

## Abbreviations

A	amaranth
ALA	α-linolenic acid
BP	blue poppy
BW	buckwheat
C	chia
FA	fatty acids
FAME	fatty acid methyl esters
H	hemp
H/H	hypocholesterolemic/hypercholesterolemic ratio
IA	index of atherogenicity
IT	index of thrombogenicity
K	canihua
L	flax
LA	linoleic acid
MUFAs	monounsaturated fatty acids
P	pumpkin
PP	plantago
PUFAs	polyunsaturated fatty acids
Q	quinoa
S	sesame
SFAs	saturated fatty acids
SM	milk thistle
SN	sunflower
